# Genetic sex separation of the malaria vector, *Anopheles arabiensis,* by exposing eggs to dieldrin

**DOI:** 10.1186/1475-2875-11-208

**Published:** 2012-06-19

**Authors:** Hanano Yamada, Mark Q Benedict, Colin A Malcolm, Clelia F Oliva, Sharon M Soliban, Jeremie RL Gilles

**Affiliations:** 1 Insect Pest Control Laboratory, Joint FAO/IAEA Division of Nuclear Techniques in Food and Agriculture, International Atomic Energy Agency, Vienna, Austria; 2 Dipartimento di Medicina Sperimentale e Scienze Biochimiche, Università di Perugia, Via del Giochetto 06122, Perugia, Italy; 3 The School of Biological and Chemical Sciences, Queen Mary, University of London, Mile End Road, London, E1 4NS, UK; 4 Maladies Infectieuses et Vecteurs: Ecologie, Genétique, Evolution et Contrôle (MIVEGEC), UMR5290 CNRS-IRD-Université de Montpellier I,Université de Montpellier II, 911 avenue Agropolis, 34394, Montpellier, France; 5 Centre de Recherche et de Veille sur les Maladies Emergentes dans l’Océan Indien, Sainte Clotilde, La, Réunion

**Keywords:** Genetic sexing, *Anopheles arabiensis*, Sterile insect technique, Dieldrin resistance, Sterility

## Abstract

**Background:**

The sterile insect technique (SIT) has been used with success for suppressing or eliminating important insect pests of agricultural or veterinary importance. In order to develop SIT for mosquitoes, female elimination prior to release is essential as they are the disease-transmitting sex. A genetic sexing strain (GSS) of *Anopheles arabiensis* was created based on resistance to dieldrin, and methods of sex separation at the egg stage were developed. The use of this strain for SIT will require sexually sterile males: useful radiation doses for this purpose were determined for pupae and adults.

**Methods:**

For the creation of the sexing strain, dieldrin-resistant males were irradiated with 40 Gy using a ^60^Co source and were subsequently crossed to homozygous susceptible virgin females. Individual families were screened for semi-sterility and for male resistance to dieldrin. For sex separation, eggs of a resulting GSS, ANO IPCL1, were exposed to varying concentrations of dieldrin for different durations. Percent hatch, larval survival, and male and female emergence were recorded. Radiation induced sterility was determined following adult and pupa exposure to gamma rays at 0–105 Gy. Mortality induced by dieldrin treatment, and levels of sterility post radiation were investigated.

**Results:**

ANO IPCL1 contains a complex chromosome aberration that pseudo-links the male-determining Y chromosome and dieldrin resistance, conferring high natural semi-sterility. Exposure of eggs to 2, 3, and 4 ppm dieldrin solutions resulted in complete female elimination without a significant decrease of male emergence compared to the controls. A dose of 75 Gy reduced the fertility to 3.8 and 6.9% when males were irradiated as pupae or adults respectively, but the proportions of progeny of these males reaching adulthood were 0.6 and 1.5% respectively

**Conclusion:**

The GSS ANO IPCL1 was shown to be a suitable strain for further testing for SIT though high semi-sterility is a disadvantage for mass rearing.

## Background

The sterile insect technique (SIT) [1,2] as part of area-wide integrated pest management (AW-IPM) programmes has celebrated many successes in suppressing, and eliminating several agriculturally and economically important insect pests in many regions of the world [3]. There is renewed interest in using sterile insects for managing endemic, as well as emerging or re-emerging vector-borne diseases, thus providing new momentum for developing SIT in the field of infectious disease control [4]. In spite of a successful SIT programme against *Anopheles albimanus* in El Salvador in the 1970s [5] most mosquito SIT programmes were either too small to demonstrate effectiveness or simply failed [6]. The development of the SIT for use in mosquito AW-IPM programmes is, therefore, in its infancy, and many fundamental components of the technique still need to be developed, validated and optimized. These include aspects of the mass-rearing of the vectors in question, the quality of the sterile males produced, and methods of handling, transporting and releasing the sterile insects within the targeted geographic region [7].

One of the many essential steps in mass production of mosquitoes for the SIT is the elimination of females, since even sexually sterile females can transmit disease pathogens. As the manual separation of the sexes based on their morphology is time and labour-intensive and with some risk of error, genetic sexing strains (GSS) based on an artificially induced sex linkage of a selectable marker are required [8].

In recent years, novel strategies for genetic sexing have been developed involving genetic modification (GM) through germ-line transformation, including systems involving testes-specific expression of enhanced green fluorescent protein [9] and a tetracycline repressible dominant lethal [10]. Various arguments are routinely made to promote the merits of individual systems [4], but leaving aside the debate on field release of GM mosquitoes, it is apparent that even the most sophisticated of novel approaches suffers some disadvantages and there remains considerable scope for conventional non-GM systems [11].

The classical approach for creating a GSS is to link a conditionally lethal allele to the Y chromosome through irradiation-induced chromosome rearrangements [8]. This is technically a genetic modification, but does not require the introduction of foreign DNA via modern biotechnology: the resulting organisms are not considered GM. Most systems previously developed in mosquitoes have been based on genes conferring insecticide resistance, where the male is heterozygous for resistance by virtue of a Y-translocation, whereas females are homozygous susceptible. Resistance to dieldrin (*Rdl*), an insecticide that blocks γ-aminobutyric acid receptors inhibiting transport of chloride ions, is the locus of first choice for *Anopheles arabiensis* for the following reasons: no other conditional lethal beside insecticide resistance has been identified in this species. The resistance is due to a single amino acid substitution in the target site [12] and can be easily detected by the PCR [13]. Both dominant and semi-dominant alleles have been identified [14], allowing homozygous susceptible and heterozygous resistant insects to be easily distinguished by a discriminating dose of insecticide in larvae and adults. The use of dieldrin for insect control has been banned since the 1970s, so the accidental introduction of resistance into mosquito populations is only important if cross-resistance becomes an issue. As dieldrin resistance is already widespread in mosquito populations and in some cases remains high [15], it is unlikely that other GABA-gated chloride channel antagonists, such as fipronil, will be used for mosquito control. However, there is evidence that fipronil can still be effective against insects carrying *Rdl*[16].

GSSs based on dieldrin resistance have been produced in the past for the experimental organism in this study, *An. arabiensis*[17] and its sibling species, *Anopheles gambiae*[8], but the strains no longer exist, so it was necessary to attempt the creation of a new strain for programmes supported by the International Atomic Energy Agency (IAEA). Previously, the necessary chromosome translocations were created with relative ease in *An. arabiensis*, with only 60 semi-sterile families screened [18]. In contrast, 216 *Anopheles stephensi* semi-sterile families were screened to recover the GSS [19].

Advantages of an *Rdl*-based GSS are that females can be eliminated at an early life stage with minimal handling, ensuring that mass production costs are low and that males of optimal quality are produced. At the same time, a stable inbreeding GSS strain can be reared under standard conditions also ensuring reduced costs. Lines and Curtis [17] demonstrated that elimination of females from the previously created *An. arabiensis* GSS could be effectively achieved by exposure of first instar larvae to dieldrin, and despite approximately 1% recombination, the strain was maintained with minimal additional selection. The creation of a new *An. arabiensis* GSS strain is reported here, but with a lower level of recombination. The strain has been used to demonstrate the separation of the sexes in which eggs, rather than larvae, are exposed to dieldrin. This not only eliminates the need to mass rear female larvae, but also greatly simplifies the separation step reducing handling of the insects and requirements for materials and equipment. Because, an SIT project requires that the released males be sterile and ANO IPCL1 is intrinsically semi-sterile, the cumulative effect of semi-sterility and induced sterility of the GSS males through irradiation is reported.

## Methods

### Mosquito stocks and rearing

Two pure-breeding stocks of *An. arabiensis* were used for creation of the GSS and other experiments. Both strains and details of their characteristics are available from the Malaria Research and Reference Reagent Resource Center under the numbers indicated. The SENNAR strain (MRA-334) contains a semi-dominant allele for resistance to dieldrin [12] and DONGOLA (MRA-856) contains only the dieldrin-susceptible allele. Although the formal symbol for dieldrin resistance is *Rdl*^R^, it will be referred to as the homozygous resistant, susceptible and heterozygous individuals as RR, SS and RS respectively. The GSS described in this manuscript has been maintained since 2008 in the Insect Pest Control Laboratory (IPCL) of the FAO/IAEA Agriculture & Biotechnology Laboratories, Seibersdorf, Austria. All strains were reared in a climate-controlled room maintained at a temperature of 27 ± 1 °C and 60 ± 10% relative humidity. The light regime was LD 12:12 h photoperiod, including dusk (1 h) and dawn (1 h). Larvae were reared in plastic trays (40 x 29 x 8 cm) at a density of approximately 500 first instar larvae (L1) per tray that contained ± 1.5 L of deionized water. Larvae were fed a diet of finely ground (224 μm-sieved) Koi Floating Blend® (Aquaricare ®, Victor, New York, USA, no longer available). Pupae were collected and placed in small plastic cups inside a fresh adult cage for emergence. Adults were kept in standard 30 cm cubic insect cages (Megaview Science Education Services Co, Ltd, Taiwan) and continuously supplied with 10% [w/v] sucrose solution with 0.2% methylparaben [20]. Females were blood-fed weekly on de-fibrinated bovine blood using the Hemotek feeding apparatus (Discovery Workshops, Accrington, Lancashire, UK). Gravid females were allowed to oviposit in plastic cups with black lining containing a wet sponge over which a filter paper was placed. Eggs were collected from individual females by placing them in a plastic medicine vial lined with filter paper and plugged with a cotton ball. For egg hatching rates, the filter paper was removed and examined under a dissecting microscope.

### Dose response of larvae

A dieldrin (291218, Sigma-Aldrich, St. Louis, MO, USA) 1,000 ppm stock solution was prepared in acetone, and all further dilutions were prepared from this. The original selection and confirmation of the resistance status of DONGOLA and SENNAR was performed by exposure of batches of 50 L3 and L4 larvae to 100 ml of dieldrin solutions (in plastic cups) of various concentrations ranging from 0.001 ppm to 10 ppm for 1 h at room temperature (approximately 25 °C). Any larvae that pupated within 1 h after the end of the dieldrin exposure were discarded, as pre-pupae are more resistant than earlier stages (data not shown). In addition to the pure-breeding susceptible DONGOLA and resistant SENNAR strain, heterozygous F1 larvae were created by crossing these strains. They will be referred to as F1 or heterozygotes.

### Creation of the GSS ANO IPCL1

Late pupae of the resistant (SENNAR) and susceptible (DONGOLA) strains were separated into males and females based on genital morphology and placed in holding cages for emergence. About 100 SENNAR males (<24 hours post emergence) were irradiated with 40 Gy using a cobalt-60 (^60^Co) source (Gammacell220, MDS Nordion, Ottawa, Canada) [21] and crossed to about 200 homozygous susceptible virgin females. The F1 males were then backcrossed to susceptible virgin females *en masse*. Females were blood-fed and placed in a holding cage for two days. For each screening round, 60 to 100 single females were placed in 2.5 x 7.5 cm glass flat bottom vials, the bottom two thirds of which was lined with filter paper and sealed with a cotton wool plug. Distilled water was added to about one third of total volume. The backcross and egg collection procedure was repeated two or three times for each of three irradiation experiments conducted.

The eggs were allowed to hatch within the vial. One day after the first larvae were observed, the empty egg cases and unhatched eggs were counted under a dissecting microscope. In the latter, where possible, the presence of an eyespot was looked for to confirm embryonic death. Only semi-sterile (<50% hatch) lines were maintained. Three approaches were taken to screening with dieldrin depending on the numbers of larvae in each line. Most lines were screened by exposing batches of 25 or fewer fourth instar larvae to 0.2 ppm dieldrin in 150 ml of distilled water in standard 210 ml plastic cups. Occasionally, a line was inbred and the test postponed to the next generation on the grounds that a promising line would show very little recombination. The third approach used, again rarely, was to expose adult males after mating to standard WHO 0.4% dieldrin papers. Only lines showing a markedly higher than expected survival of males were maintained for further analysis, which involved out-crossing resistant males to DONGOLA females. The karyotype of the finally selected strain (ANO IPCL1) was determined by examination of salivary gland chromosomes by a method described by Cornel [22].

### Routine GSS strain purification

To avoid the accumulation of undesirable recombinants (dieldrin-resistant females and males that carry the dieldrin-resistance allele in repulsion to the aberration), a pure stock was maintained by regularly out-crossing dieldrin-resistant ANO IPCL1 males to virgin DONGOLA females in a three-step process: 1) larvae of the most recently back-crossed ANO IPCL1 were exposed to 0.1 ppm dieldrin solution for 1 h and surviving (resistant) males were kept. Ten crosses were set up in small cages, each containing three resistant males and 10 virgin DONGOLA females. Egg batches were collected *en masse*, hatched and the larvae were exposed to dieldrin as described above. Entire batches of progeny containing any females were discarded and the remaining batches pooled; 2) with these males, another 10 crosses were then set up as stated above. Again, eggs were collected and larvae treated. Those batches containing no females were kept and pooled; and, 3) 100 of these males were then crossed with approximately 300 virgin DONGOLA females. Cages were kept at densities no higher than approximately 400 adult mosquitoes. The routine mode of maintaining purity of the stock repeated the last two steps, in which males surviving dieldrin treatment are backcrossed to virgin DONGOLA females. This should be done every generation to maintain a pure colony.

### Effects of dieldrin exposure on GSS eggs

To determine the effects of dieldrin exposure on GSS eggs, females of ANO IPCL1 were blood fed, and oviposition cups were placed in the cage overnight and removed the following morning (aged ≤ 12 h). The eggs were concentrated by rinsing them off of the filter paper into plastic cups lined with filter paper, to which they adhere. The eggs were counted and separated into batches of 400–600 eggs per exposure tube (made of plastic, 2 cm in diameter, the bottom of which was sealed with fine netting). These tubes allow simple and rapid exposure and rinsing of batches of eggs. The tubes containing the eggs were then placed into 50 ml of 0.5, 1, 2, 3, 4 and 5 ppm dieldrin at a constant temperature of 25 °C for 1, 2, 6, or 24 hours. After exposure, the eggs were collected and rinsed before placing them into white cups lined with filter paper containing de-ionized water and 640 μl of 1% FAO/IAEA larval diet, consisting of 0.1 mg of bovine liver powder, 0.1 mg of tuna meal and 92 μg of Vanderzant Vitamin Mix mixture per larva per day [23]. The hatch rates were observed under a dissecting microscope, and the number of L1 larvae noted. Pupae were collected by pipetting once daily and transferred to emergence tubes (BioQuip Products Inc. 2321 Gladwick Street, Rancho Dominguez, CA 90220, USA). The number of emerged male and female adults was recorded. Adults that eclosed incompletely or were unable to fly were counted as dead.

### Effects of temperature on dieldrin treatment efficacy

ANO IPCL1 eggs collected as stated above were exposed to 1, 2 or 3 ppm dieldrin for 2 h at 25 °C (ambient temperature in the treatment laboratory) or 30 °C in a water bath (TECHNE, TE-10A, Bibby Scientific Ltd., Stone, Staffordshire, ST15 0SA, UK). There were three replicates for each treatment. Effects on hatch rates, number of surviving males and females were observed.

### Effects of egg age on treatment efficacy

It was hypothesized that because fresh eggs are white and soft and progressively melanize and sclerotize that their permeability to dieldrin would change with age. Therefore, eggs were collected at intervals of less than two hours to ensure a defined narrow age range. All eggs were still white or whitish yellow in colour when collected. These eggs were then exposed to 1 ppm and 3 ppm dieldrin solutions when “young” (<12 hours old) and “old” (≥24 hours old). Treatment was stopped and the batch discarded if eggs began to hatch.

### Radiation-induced sterility

ANO IPCL1 pupae (n = 50) were collected within a six-hour interval after pupation and irradiated 20 h later. They were placed on a wet net, in a 4 cm diameter cup at the centre of the irradiation chamber. Approximately 15 hour-old adult males (n = 50) were placed in a 4 cm diameter container using a buccal aspirator without anaesthesia. The container was then put in contact with ice for several minutes to chill the males before irradiation. The container was maintained on ice to keep the males immobilized during the irradiation process. All treatments were left on ice for the maximum irradiation time so that the chilling effect on all groups would be similar. Pupae and adults were exposed to gamma rays emitted by a ^60^Co source at 0, 60, 75, 90, and 105 Gy (dose rate ca. 9 Gy/min). The Gafchromic HD-810 film (International Specialty Products, NJ, USA) dosimetry system was used to measure the dose received by the lot; three dosimeters were included with each lot of insects and read after irradiation with a Radiachromic reader (Far West Technology, Inc., California, USA). Males were offered virgin females from the wild strain DONGOLA in a 1:1 ratio. Virginity of females was ensured by separating them from males at the pupal stage. After five days, females were blood-fed with human blood from a volunteer and allowed to oviposit in individual tubes. Egg hatch rates were then recorded, as well as the number of L1 alive. For the treatments 60, 75, 90 and 105 Gy, all L1 were transferred to Petri dishes for rearing. Density was less than two larvae/ml and feeding was standardized (0.2 mg of diet/larva/day) for all treatments. The number of pupae and emerging adults was recorded for each family.

### Statistics

The analyses were conducted using R [24]. Results of resistance assays were analysed by logistic regression using the DR routines of the R statistics package. The dieldrin-induced mortality on eggs, larvae and adults was corrected from the control mortality levels. The female or male production rates were calculated as the number of emerged adults out of the total number of eggs. Egg eclosion rates, mortality rates, and adult production rates were square-root-transformed to achieve normal distribution; ANOVA (*P* < 0.05) and Tukey Post-hoc tests were used to compare treatments.

Egg hatch rate data were square-root-transformed and compared between treatment using ANOVA and Tukey Post-hoc tests. Within one treatment, Kruskall-Wallis rank sum tests were used to compare the proportions of hatching eggs, L1 or emerging adults resulting from the progeny of irradiated males (*P* < 0.05).

## Results

### Dose response of larvae

**Figure 1:**
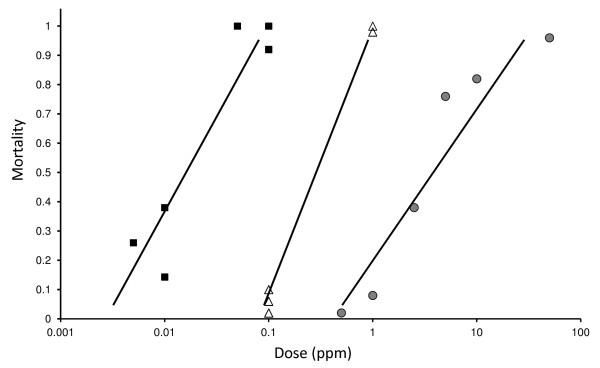
**Larval dose–response curves.** Left to right are DONGOLA (SS), F1 hybrids and SENNAR (RR). The dose 0.1 ppm dieldrin for 1 h was selected to eliminate susceptible larvae based on these analysis and was used for selection of the GSS.

The three dieldrin genotypes were easily distinguished by dieldrin exposure in the larval stage. Briefly, all SS larvae are susceptible to 0.1 ppm dieldrin and RS individuals survived doses up to 1.0 ppm (Figure 1) for 1 h. RR individuals survived doses exceeding 1.0 ppm. On the basis of these susceptibilities, a discriminating dose of 0.1 ppm was chosen to select RS and RR individuals and to kill SS larvae.

### Creation of the GSS ANO IPCL1

Approximately two-thirds of 750 females from the three irradiation experiments oviposited. As the focus was the isolation of a useful GSS rather than an evaluation of the procedure, investigations of individual lines were minimal. The cut-off for the initial screening for semi-sterility was applied at 50% hatch or less; however, lines in the higher end of this range rarely showed semi-sterility in the next generation, a characteristic that would be expected for an appropriate chromosome rearrangement. Three lines in this category were found amongst 19 initially classified as semi-sterile from three rounds of screening in the second irradiation experiment. A further two lines did not survive rearing in sufficient numbers to maintain the lines. Ten were discarded after first, or second, generation dieldrin assays. Amongst a few lines that had not yet been fully evaluated was 5–33. The initial bioassay was performed on 10 adult males after mating, only one died. It was particularly difficult to amplify the line to obtain sufficient numbers for bioassays and ensure its survival, since egg yield and egg hatching were low. Line 5–33 (ANO IPCL1) was kept for about nine months without selection, but with an occasional supplement of virgin DONGOLA females. A bioassay was then conducted on 500 early fourth instar larvae using a concentration of 0.1 ppm under standard conditions. Mortality after the 24-h holding period was 48%. Only two females were obtained amongst the survivors indicating a recombination frequency of 0.4% or less. A subsequent experiment in which approximately 3,000 first instar larvae were exposed *en masse* in one large tray to 0.1 ppm dieldrin resulted in no female survivors. The strain shows high semi-sterility, with an average percent hatch of eggs at 26.7% regardless of the data collection method (family data, 95% CI = 0.015, n = 220; *en masse* egg-collection data 95% CI = 0.023, n = 34).

**Figure 2:**
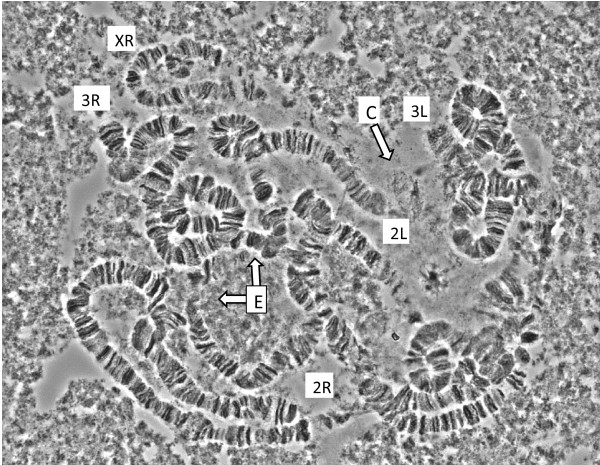
**Karyotype of the GSS.** C refers to the putative chromocenter, and E the vicinity of the exchange. Labels for chromosome arms have been placed in the vicinity of their telomeres. Because only one copy of the X chromosome is present in males, chromosome XR is expectedly narrower than the autosomes.

The karyotype of the GSS is complex, a finding consistent with the low hatching rate: neither the X nor 3 L chromosome is involved (Figure 2). Determining the exact positions of the break points was not possible because of the complexity of the translocation and the resulting difficulties to obtain properly spread chromosomes. Furthermore, the only existing photographic chromosome map [25] has incorrect arm and band assignments (V Petrarca, pers. comm.). The best interpretation is that there is a peri-centric inversion including much of chromosome 2R with a break at 9A, and on 2L in division 22. The breakpoint may be common with a Y-chromosome translocation having a breakpoint basal on 3R. It is quite possible that the aberration is even more complex. For example, an alternative explanation is that it contains a chromosome 2–3 translocation. The dieldrin resistance allele in on chromosome 2L in division 22A [12], i.e. approximately one-third of the arm length from the centromere, a location that would be well within the putative peri-centric inversion.

### Effects of dieldrin exposure on ANO IPCL1 eggs

Two manifestations of dieldrin toxicity were expected as a result of egg exposure: failure of larvae to hatch and delayed mortality during the later stages of development. Several preliminary observations were made to determine the treatment parameters that would be most effective. To ensure that the acetone concentration of the solutions was not affecting hatch rates or larval survival, <12-h old eggs were exposed to acetone solutions for 1 h at concentrations up to 1%, the highest concentration used in these experiments. No increases in larval mortality or change in hatch rates were observed.

**Figure 3:**
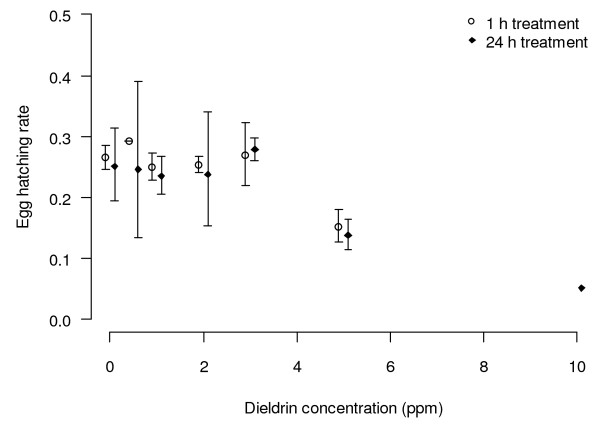
**Effects of dieldrin treatments on egg eclosion of ANO IPCL1.** Mean hatch rate (± CI) following exposure for 1 h or 24 h treatments.

Non-treated ANO IPCL1 eggs hatched an average rate of 25.7 ± 0.9%. The mean hatch remained between 22 and 29% up to a concentration of 3 ppm, then dropped to 14.4 ± 0.9% (mean ± SEM) at exposures to 5 ppm dieldrin (Figure 3). No statistically significant differences in hatching rate were observed between control, 0.5, 1, 2 and 3 ppm dieldrin solution treatments for any of the treatment durations. However concentrations of 5 and 10 ppm significantly reduced the hatching rate (F_7, 76_ = 19.41, *P* < 0.001). No interaction was observed between the time and concentration of dieldrin treatment (F_13, 76_ = 0.64, *P* = 0.81). For each dose, the duration of exposures of 1, 2, 4, 6, or 24 h had no effect on hatch rate (F_4 76_ = 2.31, *P* = 0.07), thus all data from a same concentration were merged for the next analyses. It is suspected that some susceptible females died shortly after hatching as the numbers of L1 larvae counted were far lower than what would be expected based on the hatch rate.

**Figure 4:**
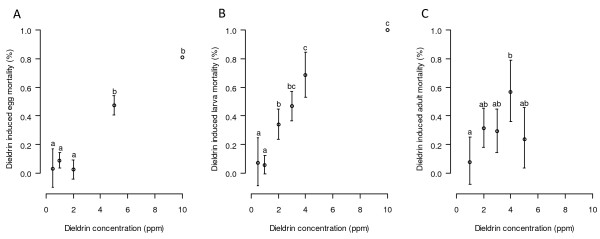
**Dieldrin-induced mortality on eggs (A), larvae (B) and adults (C).** Mean mortality rate (± CI ) for different concentrations of dieldrin. Different letters indicate a significant difference (P < 0.05).

In control treatments the survivorship from hatched eggs to L1 larva was 92.1 ± 2.3% but only 66.8 ± 5.3% of the hatched eggs survived to adulthood. The mortality rates of eggs (Figure 4, panel A), from hatched eggs to larvae (Figure 4, panel B) and from hatched eggs to adulthood (Figure 4, panel C) were corrected from the control values for the dieldrin treated groups. The dieldrin induced mortality increased with the dieldrin concentration for the various developmental stages. Although no females appeared after treatments at 5 ppm dieldrin, the number of males obtained was too small for this concentration to be useful. Concentrations greater than 2 ppm dieldrin induced an increase of 35 ± 5% of larval mortality and 33 ± 6% of adult mortality as compared to the normal mortality of the untreated batches.

**Figure 5:**
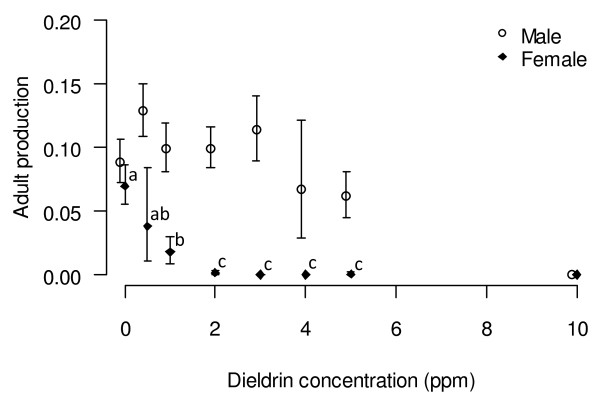
**Efficiency of dieldrin treatments on male emergence and female elimination.** Adult emergence (calculated as the rate of adults emerged out of the total number of eggs ± CI) of male and female adults out of the initial number of eggs as a function of dieldrin concentration.

Assuming an equal sex ratio with a natural fertility of 27%, this strain can produce a maximum of 13% of males from the initial number of eggs. This production fluctuated between 9 and 13% in treatments up to 3 ppm; and decreased to 6.4% when treated at 4 and 5 ppm (Figure 5). However there was no significant difference between all these treatments (F_6, 93_ = 2.19, *P* =0.051).

Five of six egg batches treated at 0.5 ppm yielded some females, a result similar to batches treated at 1 ppm where 16 of 27 batches produced a mean 2.9 ± 0.6% of females from the initial number of eggs. Significant differences were observed between the treatments 0, 0.5, 1 ppm and the higher concentrations (F_6, 93_ = 28.7, *P* < 0.001). At 2 ppm, the production of females was significantly reduced to 0.4%; of 16 batches only three yielded ≥ 1% females. (These females, when selected on 1 ppm dieldrin paper survived, suggesting that they were most likely dieldrin resistant). At 4 ppm, none of the egg batches yielded any females, however the number of males obtained fell below 10% of the original number of eggs indicating that delayed mortality was occurring even among the RS individuals.

### Effects of solution temperature and egg age at treatment on male and female emergence and egg hatch rate

The egg-hatching rate was significantly lower when treatment occurred at 30 °C as compared to 25 °C (F_1, 16_ = 11.45, *P* < 0.01); control batches hatched at 24.6% at 30 °C against 26.4% at 25 °C. No interaction between concentration of dieldrin and treatment temperature was detected. Temperature did not affect the number of adult females emerging after treatment (F_1, 16_ = 0.33, *P* = 0.58).

**Figure 6:**
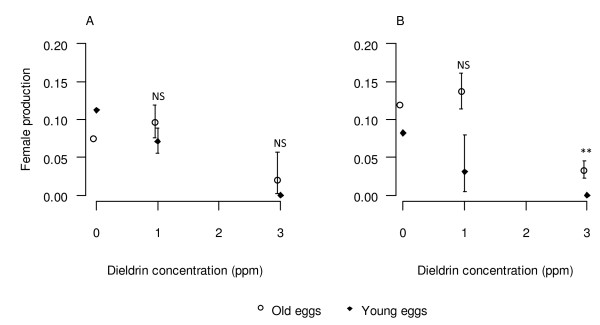
Effect of egg age on dieldrin toxicity. Female emergence (calculated as the rate of females emerged out of the total number of eggs ± CI) after treatment of 1 h (A) or 6 h (B) with old (> 24 h) or young (aged ≤ 12 h) eggs.

The age of eggs when treated had a significant effect on the production of females (F_1, 16_ = 42.0, *P* < 0.001), however significant interactions were found between age and concentration (F_2, 16_ = 4.19, *P* < 0.05) and between age and time of treatment (F_1, 16_ = 6.99, *P* < 0.05). Of the 9 batches of young eggs treated at 3 ppm (for 1, 6 and 24 h treatments), all yielded males only (Figure 6). When more mature eggs (12 hrs or older) were treated, all three batches treated yielded females. It is therefore important to treat the eggs while the eggs are less than 12 hrs old, when the treatment is more effective in killing females.

### Radiation induced sterility of ANO IPCL1

ANO IPCL1 males were irradiated at various doses either as late pupae or <15 h old adults. The mean natural fertility of the two control groups in these particular experiments was 29.7 ± 3.0% (Table 1). At a dose of 75 Gy ca. 95% sterility was observed when considering the egg hatch rate: the fertility did not differ significantly over 75 Gy for pupal irradiation and 90 Gy for adult irradiation. The reduction of fertility that could be attributed to gamma irradiation was similar for the pupal and adult stages, and they were similar to those observed on the wild *An. arabiensis* DONGOLA strain [26] and to those reported by Helinski *et al*[21] with the *An. arabiensis* KGB strain, originating from Zimbabwe. The survival of the progeny was followed until adult emergence. The mortality between hatched eggs and L1 for un-irradiated ANO IPCL1 males’ progeny was 20 ± 3%. Progeny larval mortality increased with the radiation dose received by the male. When male pupae were irradiated at 75 and 105 Gy, the progeny larval mortality rate was respectively 52 and 64%, and for adult irradiated at 75 and 105 Gy, it was 38 and 57% respectively. When adults were irradiated at 105 Gy, 96% of them had no viable adult progeny; only one emerged adult was found in two out of 29 broods. When males were irradiated as adults at 90 Gy, 78% of ANO IPCL1 did not produce any offspring that reached adulthood; the remaining 22% of the males produced only one adult offspring. When males were irradiated as pupae at 75, 90 or 105 Gy, more than 80% of them had no or only one surviving offspring. When considering the fertility as the proportion of eggs resulting in adults, the mean fertility was 3.1 ± 0.6% and 2.1 ± 0.4% respectively for the pupal and adult irradiation at 60 Gy. More than 98% sterility was reached from doses over 75 Gy for both pupal and adult irradiation.

**Table 1:** Radio-sterilization of ANO IPCL1. Percentage of egg hatch, resulting in first instar larvae or in emerged adults in progeny from males subjected to different radiation doses at the pupal or adult stage.

**Dose (Gy)**	**Stage of irradiation**	**Percentage of hatched eggs**			**Percentage of 1st instar larvae**			**Percentage of emerged adults**		
		**Mean**	**SEM**		**Mean**	**SEM**		**Mean**	**SEM**	
0	Pupa	33.9	1.6	a	28.2	1.9	b			
	Adult	27.6	1.9	a	22.4	1.7	b			
35	Adult	12.9	1.2	a	9.6	1.3	b	2.4	0.8	c
60	Pupa	8.3	1.0	a	5.8	0.9	a	3.1	0.6	b
	Adult	5.8	0.8	a	3.6	0.6	a	2.1	0.4	b
75	Pupa	3.8	0.8	a	1.3	0.5	a	0.6	0.3	a
	Adult	6.9	1.1	a	3.8	1.0	b	1.5	0.5	b
90	Pupa	4.6	0.8	a	1.6	0.4	b	1.0	0.3	b
	Adult	2.6	0.6	a	0.8	0.3	a	0.2	0.1	b
105	Pupa	4.2	0.7	a	0.9	0.3	b	0.5	0.2	b
	Adult	1.2	0.3	a	0.2	0.1	b	0	0	b

Within one treatment (same dose and stage of irradiation), values followed by different letters are statistically significantly different (P < 0.05).

Emergence of adults could not be followed for control treatments.

## Discussion

For ethical and public health reasons, female mosquitoes must be eliminated from releases. There are many advantages of a GSS that allows separation in the egg stage: cost reductions in the production process can be considerable if only half of the number of larvae is cultured [3], and almost exclusively male pupae and adults are immediately available for irradiation, transport and release. Treatment at the egg stage may not only improve the quality of the ANO IPCL1 males by minimizing damage to them due to handling during the larval stages [27] and eliminating unnecessary larval culture, but sex separation becomes more practical and accurate.

To these ends, a GSS for *An. arabiensis* was created and tested and methods for exposing eggs to eliminate females were developed. GSS utilizing a selectable marker with recombination frequencies <1% have been created in mosquitoes previously: *An. gambiae*, 0.25% [8]; *An. arabiensis* (no specific value given but well below 0.1%) [28]; *An. albimanus,* 0.3% [29]; *Anopheles quadrimaculatus,* 0.02% [30]; *An. stephensi,* 0.3% [19]; *Anopheles culicifacies* < 0.02% [31]. All of these used either malathion, dieldrin or propoxur as the selectable marker for obvious reasons: resistance is relevant to public health and is often quickly selected in wild populations and easily identified in stocks.

GSS creation depends on fortuitous isolation of aberrations that suppress recombination between the selectable marker and the Y chromosome. In the case of this GSS, the number of families screened was unusually large. In remarkable contrast, only 18 families were screened to identify a previously created GSS for *An. arabiensis*[28] which was also based on dieldrin resistance.

The conditions for egg exposure to dieldrin do not appear to be stringent. The ideal dieldrin concentration to eliminate all females during the egg stage lies between 2 and 3 ppm for a duration from 1–6 hrs in the temperature range of 25-30 °C. While these experiments demonstrated that exposing eggs when young is important, the degree of latitude that is possible is not known yet. Further trials are needed to assess the efficacy of the treatments when treating larger quantities of eggs being prepared for mass releases. Additional refinements to the technique such as the quantification of eggs volumetrically would greatly enhance efficiency and accuracy when treating larger quantities on a daily basis. Such methods applied to a GSS of *An. albimanus*[27], including egg treatment, greatly facilitated production of this species, and similar benefits are expected for *An. arabiensis*.

The ANO IPCL1 shows high intrinsic sterility of 73%, which results in the production of a maximum of 13% males from the total number of eggs. This puts great pressure on the brood stock production level for mass production, but this should not be an insurmountable obstacle. The MACHO GSS strain of *An. albimanus* showed sterility of 50%, and yet they were able to produce one million sterile males per day [27]. Balancing this limitation is the potential advantage of fairly high sterility inherited from GSS males by any male progeny in the field. This allows for the possibility that the irradiation dose can be reduced to attain the same level of population suppression that would require greater irradiation when using a GSS with higher fertility. A reduced dose generally improves competitiveness*.* Indeed, as part of an integrated pest management approach, release of a semi-sterile strain subjected to radio-sterilization has been considered: this is known as the “Combi-Fly concept” [32,33]. Full radio-sterilization of wild strains usually leads to a lower competitiveness of males as compared to non-irradiated ones [34]. Irradiation produces dominant lethals, which lead to a dose-dependent lethality among the offspring. This death would occur predominantly at the very early stage of embryonic development: Laven and Jost [35] reported that embryos could not be detected in most of the non-hatching eggs fathered by irradiated male *Culex pipiens*. However, irradiation affects similarly normal sperm and sperm carrying a translocation, hence the fully sterilizing dose should not differ greatly between a wild strain and a GSS [36]. As a matter of fact, the radiation-induced sterility in ANO IPCL1 showed the same rate of increase as the wild strain DONGOLA [26]. However, a greater difference between the wild and the ANO IPCL1 strain appears when looking at the survival of the progeny. An average of 19.7% first instar larvae died soon after hatching in the progeny from ANO IPCL1 un-irradiated males and this mortality rate increased greatly with the radiation dose. What really matters in the release of sterile males is the final number of adults that would result from the mating of wild females and sterile males. Thus, the sterilizing dose should be chosen accordingly and sterility rates of genetic sexing strains should not be evaluated only as the egg hatch rates but rather as the proportion of eggs leading to adults. Considering this, ANO IPCL1 shows > 96% sterility at a radiation dose of 60 Gy and a dose of 75 Gy appears sufficient to lead to > 98 % sterility. It was reported as well for other GSSs that, in addition to a reduced egg hatch, males usually sire progeny with a reduced survival rate during the later developmental stages [37]. This lethality could be explained by the presence of triplication carrying individuals that resulted from adjacent segregation during meiosis in the male parent [34]. The chromosomal study showed that the translocation was complex in this GSS; this is consistent with the high lethality observed in the various stages of the progeny fathered by ANO IPCL1 irradiated males**.** This later mortality brings the advantage of maintaining larval competition in the breeding sites and thus maintain a low wild larval survivorship through density-dependence effects [38].

Though genetic recombination in the ANO IPCL1 occurs at a low rate, it requires management. Leaving recombinants unchecked runs a risk of deterioration of the strain. Therefore, there is a need to periodically purify the strain by keeping a homozygous susceptible stock to outcross ANO IPCL1 males to on a regular basis. At this time, there is no data demonstrating the accumulation rate of breakdown progeny of the strain.

While insecticide-resistance alleles are widely available, systems based on chemical toxicity can be disadvantageous for several reasons: contamination of the susceptible rearing colony is always an immediate danger; use of insecticide requires that residues, contamination, and waste management are all issues that must be dealt with appropriately. Furthermore, dieldrin solutions become less potent once used [39]. It is presumed that the dieldrin molecules are absorbed and/or adsorbed by eggs as well as onto the surfaces of containers in which the treatments are performed. Therefore the solutions should not be reused for consecutive treatments.

In order to avoid the disadvantages of having a toxicant in the insectary, it is desirable to develop a sex separation system that relied on a physical selection treatment such as one based on a temperature sensitive lethal mutation. This has been accomplished in *Culex tritaeniorhynchus*[40] similar to the system used for medflies in mass production facilities [37]. The *Cx.* temperature-sensitive lethal isolation depended on an array of genetic markers that were available during the heyday of classical mosquito genetics, but these are no longer extant for any mosquito species, so the difficulty of isolating additional lethals should not be underestimated.

There may be an intrinsic loss of vigour related to the dieldrin resistance gene. The biological quality of RR males and females of *An. gambiae* Giles and *An. stephensi* Liston have been compared to RS and susceptible SS males and females [36,41]. It was found that the females of resistant strains were less responsive to oviposition stimuli, produce fewer eggs per unit of blood, fly less when seeking hosts or oviposition sites and respond slower to simulated predators. The males were generally less successful in competing for females. It is thought that perhaps the mating success of RR males was poorer because of their reaction to female swarms (as to predator movements) was generally slower. These results should be considered carefully as there was no attempt to distinguish strain from resistance gene effects. However, in the light of other findings, the general fitness and quality of ANO IPCL1 must be scrutinized with a series of experiments to ensure that there are not prohibitive reductions in competitiveness.

## Conclusion

The GSS reported here provides a suitable strain to proceed toward releases and has been used in a small scale field release in northern Sudan primarily concerned with evaluating logistics. Its performance characteristics will have to be tested in detail, but mating competition studies in large cages and semi-field conditions using sterilized males from the susceptible parental strain were very encouraging [42]. It is certain that without a GSS, releases on an operational scale cannot occur. While transgenic methods for sex-separation [43] and sterilization [44] are being developed, it is not yet clear that their potential advantages will be realized.

## **Competing interests**

The authors declare that they have no competing interests.

## **Authors’ contributions**

HY carried out the experiments and collected the data for the egg-dieldrin exposures, and drafted the manuscript. JG as project supervisor, oversaw the design of the egg- exposure study, as well as the progress of the manuscript, and managed the collaboration between authors. CAM together with SMS created the GSS ANO IPCL1 and provided the manuscript for this section. MQB performed the dose–response assays, provided essential expertise for the egg exposure experiments and contributed greatly to the development and editing of the manuscript. CFO designed and performed the radiation induced sterility study and completed the statistical analysis for this study, as well as for the egg exposure experiments in this manuscript. All authors read and approved the final manuscript.
